# Traditional medicine trade and uses in the surveyed medicine markets of Western Kenya

**DOI:** 10.4314/ahs.v22i4.76

**Published:** 2022-12

**Authors:** Willy Kibet Chebii, John Kaunga Muthee, James Karatu Kiemo

**Affiliations:** 1 Department of Earth and Climate science; Department of Plant Science and Crop Protection; University of Nairobi, Nairobi, Kenya; 2 Department of Earth and Climate science; Department of Clinical Studies; University of Nairobi, Nairobi, Kenya; 3 Department of Earth and Climate science; Department of Sociology and Social work, University of Nairobi, Nairobi, Kenya

**Keywords:** Traditional Medicine, Common disease treated, Medicine markets

## Abstract

**Background:**

There exist vast traditional medicine and herbal remedies prescribed for diseases and socio-cultural ills that are sold in local medicine markets.

**Objectives:**

To assess the common traditional medicine traded in the local medicine markets and used for treating common diseases.

**Methods:**

The study was carried out in nine purposively selected medicine markets spread out in seven administrative counties of Western Kenya. Purposive sampling with elements of snow ball method was employed in the identification of willing respondents. In addition, face to face interviews were conducted with the aid of a pre-tested semi-structured questionnaire that sought to extract a targeted and expertise information from the respondents.

**Results:**

The survey recorded 45 commonly traded plant families composed of 78 genera and 87 medicinal plant species. Meliaceae, Apocynaceae and Fabaceae were leading plant families whereas *Trichilia emetica, Azadirachta indica, Dregea schimperi and Aloe spp.* were commonly traded.

**Conclusion:**

Traditional medicine traded in the local medicine markets continue to play a significant role in the treatment of common diseases. Frequently traded medicinal plant species should be prioritized for conservation.

## Introduction

Traditional medicine (TM) is a subset of ethnomedicine and it encompasses natural local resources including medicinal plants in the treatment of various diseases and socio-cultural ills or syndromes. Up to 80% of people in the developing world use TM as a source of primary health care due to a wider belief that they are affordable, safe, easy to use, involve simple market prescriptions, ease of access by urban and per-urban populations. The high costs of modern medicine limit access to allopathic medicine.

The resurgence of interest in TM has been largely attributed to the challenges experienced in the treatment of chronic diseases using allopathic medicine, for instance, diabetes, hypertension, HIV/AIDS, and cancer. TM trade is key in the promotion of socio-economic, cultural and environmental benefits and also in supporting lives and livelihoods of the practitioners, collectors and major stakeholders in the sector. Few TMPs of the Western Kenya rely wholly on the TM trade, some ply the trade as an additional source of income. The earnings are generally modest and may not cover most of their financial needs. Some TMPs practice TM trade as a family business since it is largely inherited from close family relatives. TM trade also serves as a source of revenue to the county governments through collection of market levies. It has been documented that only seven (7) out of thirty (30) most frequently species in the Northern Kenya has been estimated to account for an annual volume of 5,500 kg valued at an annual retail value of US$ 25,900.00. Regionally, Ghana and Gabon have registered impressive annual retail values of US$ 7.8 million and US$ 1.5 million respectively^1^. Therefore, the economic importance of trade in medicinal plants cannot be underestimated. However, this resurgence of trade in TM is hampered by the modern and ever changing human lifestyles^2^,^3^.

There exists a myriad of TM used in the treatment of diseases and socio-cultural ills, most of which are not documented. From the market surveys, disease identification and terminologies are mostly referred in local languages and are translated into their equivalent modern disease terminologies in order to clearly understand the nature and complexity of the TM trade^4^. Herbal remedies are mostly consumed to promote good health and well-being. Consumption of these herbal remedies at an individual level has been linked to socio-demographic factors, for instance, education and finance^5^. This study provides a documentation of the commonly traded medicine sold in the local markets and used in the treatment of common diseases afflicting the population in Western Kenya region. The study will use the Economic Botany Data Collection Standard (EBDCS) in the broad classification of diseases and also capture the specific disease citations from the TMPs^6^–^8^.

## Materials and methods

### Study area and Selection criteria

**Figure 1:**
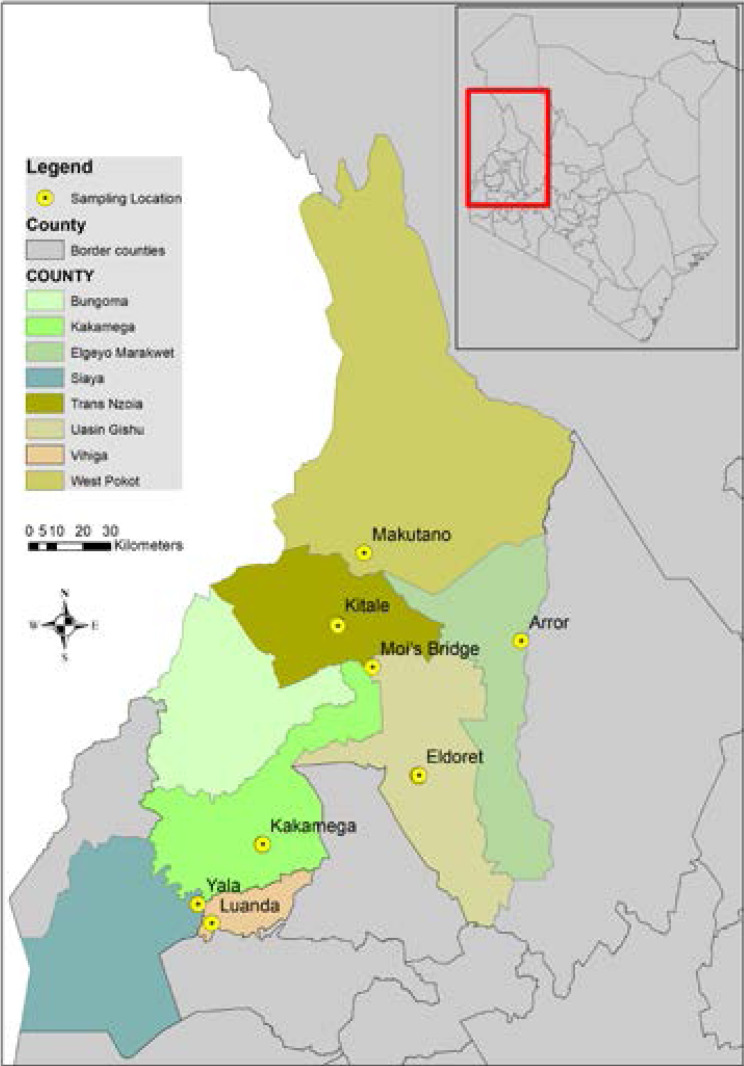
Sampled medicine markets of the Western Kenya region namely: Eldoret, Makutano, Kitale, Moi's Bridge, Arror, Kakamega, and Yala and Luanda.

The study was carried out in nine (9) selected medicine markets located in seven (7) counties in the Western Kenya region namely: Kitale and Moi's Bridge (Trans Nzoia County), Makutano (West Pokot County), Eldoret (Uasin Gishu County), Arror and Kaptabuk (Elgeyo Marakwet County), Kakamega market (Kakamega County), Luanda (Vihiga County) and Yala in Siaya County. Comprehensive description of the study area, socio-demographic, edaphic and cultural characteristics has been documented^4^. All the medicine market sites and locations were purposively selected following guidance and recommendations from Cultural Officers at the Department of Culture and professional advice from the elected officials at the National Traditional Health Practitioners' Association (NATHEPA). The sampling GPS coordinates of the medicine market locations were recorded using a Garmin etrex 20x and were used to generate a map representation of the sampled medicine markets using the Q GIS Software (Figure 1).

### Data collection

The study used a non-probability purposive or judge-mental sampling where the sample selected was knowledgeable on the set study objective, reliable, and expressed willingness to share information on their expertise. This desired sampling method is a subset of convenience sampling and is also termed an expert purposive sampling method that is objective, specific and therefore primarily targeted traditional medicine practitioners' (TMPs) plying their trade at local medicine markets. This non-probability form of sampling also provides opportunity for useful generalizations in a theoretical, analytical or logical nature^9^. In that regard, and based on the research objective, all the thirty (n=30) TM traders who were willing to share expertise information were interviewed. This was after procuring oral prior informed consents from all the willing respondents^10^. Additional oral interview approvals was obtained from the appointed medicine market leadership endorsed by the market traders. However, most medicine markets lacked an elected or appointed leadership but rather operated from an informal market arrangement setting. The study was conducted as per the set guidelines and protocols of the University of Nairobi doctoral studies https://erc.uonbi.ac.ke/ (Ref. No. UoN/ECS/9/1/21 and supported by oral prior informed consents of the willing TM traders. This was done in accordance to all social interviews devoid of human experiments as outlined by study objectives. Important inclusion and exclusion criteria observed in the research design is summarized in Table 1.

**Table 1:** Consideration of Inclusion and Exclusion criteria in the survey

Inclusion criteria	Exclusion criteria
The interviewed TMPs must be practicing in the surveyed medicine market	There will be no conditional tokenism, bribery or corruption
Willingness to freely participate in the survey	Coercion
Sharp focus on the experts and expertise information	Apprenticeship
Acquisition of an oral prior informed consent	Unwillingness to freely participate
Cultural awareness	Quackery

Face-to-face Interviews with TM traders were conducted with the aid of a pre-tested and a liberal semi-structured questionnaire that sought to extract a targeted and expertise information from the respondents^11^. The original questionnaire was written in English language and translated into Swahili language by the faculty at the Department of Linguistics, Languages and Literature based at the University of Nairobi for fieldwork purposes. The oral interviews were conducted simultaneously alongside structured market surveys and informed observations as from February 2019 to September 2019. Medicinal plant parts traded that expressed clear morphological and/or floral characters were properly identified by plant taxonomists at the University of Nairobi Herbarium (NAI). Strict emphasis was placed on the standard binomial nomenclature and herbarium techniques in handling both for fresh plant specimens and carpological materials^10^, ^12^–^16^. Due to low literacy levels of most TMPs, interviews were conducted predominantly in the Swahili national language whereas traded medicinal plants were recorded in dominant vernacular languages. All the scientific names of the medicinal plants were authenticated, validated and verified using https://www.tropicos.org/ and http://www.theplantlist.org/ online botanical databases.

### Data analysis

The importance of medicinal plant species in the sampled medicine markets was evaluated using frequency of citations or mentions by the interviewed TMPs.

Relative Frequency of Citation (RFC) was used to quantitatively determine the local importance of the identified species using the formula RFC = FC/N; 0<RFC<1 where FC represents the Frequency of Citation or mentions by each respondent and N denotes the total number of the respondents who participated in the interview. The use of FC was predominantly favoured in the survey since it positively and significantly correlates with number of uses of medicinal plants as reflected by various plant use categories^17^,^18^. IUCN status of the frequently cited medicinal plant species were assessed and placed in their respective IUCN Red List Criteria Version 3:1^19^. All the data was collated and processed in Microsoft Excel and summarized using descriptive statistics (frequency, mean, and percentages) and presented on tables, figures and graphs.

## Results

### Socio-demographic characteristics of the TM traders in Western Kenya

Women TMPs dominated the TM trade in the surveyed markets of Western Kenya and composed of 57% and the remainder were male TMPs. Overall, the average age of the TM traders was 61 years with a mean of 24 years of experience in the TM trade. The individual age of TM traders varied from 30 to 85 years with the practicing experience ranging from 2 to 48 years (Table 2). TM traders with elementary or primary level of education (n=17; 57%) dominated the TM markets.

**Table 2:** Socio-demographic, ethnic representation and distribution of the TM Traders in the selected markets of Western Kenya

County	Medicine market surveyed (*Number* *interviewed*)	Sex representation	Ethnic (Sub-tribal affiliation)
Uasin Gishu County	Eldoret (n=6)	4 Females	Kalenjin (Marakwet)
		1 Male	Kalenjin (Marakwet)
		1 Female	Kalenjin (Keiyo)
Trans Nzoia County	Kitale (n=5)	1 Male	Mijikenda/Swahili
		1 Male	Turkana
		3 Males	Luhya (Bukusu)
	Moi's Bridge (n=1)	1 Male	Maasai
West Pokot County	Makutano (n=3)	3 Females	Kalenjin (Pokot)
Elgeyo Marakwet County	Arror (n=2)	1 Female	Kalenjin (Marakwet)
		1 Male	Kalenjin (Marakwet)
	Kaptabuk (n=1)	1 Female	Kalenjin (Marakwet)
Kakamega County	Kakamega (n=5)	4 Males	Luhya
		1 Female	Luhya
Vihiga County	Luanda (n=6)	5 Females	Luhya
		1 Male	Luhya
Siaya County	Yala (n=1)	1 Female	Luo/Luhya

Only 40% of the TM traders had acquired the secondary level of education and only one practitioner had attained a tertiary education level.

### Medicinal plants frequently traded

From the identified taxa, forty-five (45) medicinal plant families were commonly traded in the surveyed medicinal markets of Western Kenya. Species comprising the Meliaceae family (n=22, 12%) dominated the medicinal markets followed by Apocynaceae (n=19, 10%), Fabaceae (n=17, 9%), Rutaceae (n=10, 5%), and Euphorbiaceae (n=10, 5%) respectively. Other traded plant families collectively represented 41% of the traded TM plant species. A total of 78 genera and 87 medicinal plant species were also recorded, with Trichilia emetica (RFC=0.37), Azadirachta indica (RFC= 0.27), Dregea schimperi (RFC=0.27), *Aloe* spp. (RFC= 0.23) and Carissa spinarum (RFC= 0.23) ranked in order of local importance (Table 3). Trichilia emetica was commonly traded in the Kakamega, Luanda and Yala medicine markets whereas Dregea schimperi was mostly traded in the Eldoret and Arror medicine markets. Prunus africana (n=4, 2%) was marketed as a cure for prostate cancer and as a natural boost for male virility. Noticeably, Tylosema fassoglense (n=4, 2%) was a dominant tuber on medicinal market displays. Notable food crop and local vegetables that were traded as medicine include the Cassava plant (Manihot esculenta), African spider flower plant (Cleome gynandra), and African Black Nightshade (Solanum nigrum). The tree growth form (46%) was the leading plant habit exploited for medicine followed by shrubs (22%), climbers (16%) and herbs (11%).

**Table 3:** The frequency of citations (FC) and Relative Frequency of Citations (RFC) of the most traded medicinal plants in the surveyed Western Kenya region

SN	Name of medicinal plant species	Frequency of citation	Relative frequency of Citation
1	*Trichilia emetica*	11	0.37
2	*Azadirachta indica*	8	0.27
3	*Dregea schimperi*	8	0.27
4	*Aloe spp.*	7	0.23
5	*Carissa spinarum*	7	0.23
6	*Erythrina abyssinica*	5	0.17
7	*Warburgia ugandensis*	5	0.17
8	*Zanthoxylum chalybeum*	5	0.17
9	*Prunus africana*	4	0.13
10	*Tylosema fassoglense*	4	0.13
11	*Bridelia micrantha*	3	0.10
12	*Capparis tomentosa*	3	0.10
13	*Clerodendrum myricoides*	3	0.10
14	*Ekebergia capensis*	3	0.10
15	*Maerua decumbens*	3	0.10
16	*Salvadora persica*	3	0.10
17	*Terminalia brownii*	3	0.10
18	*Ziziphus mauritiana*	3	0.10
19	*Cucumis aculeatus*	3	0.10
20	*Solanum incanum*	2	0.07

**Figure 2:**
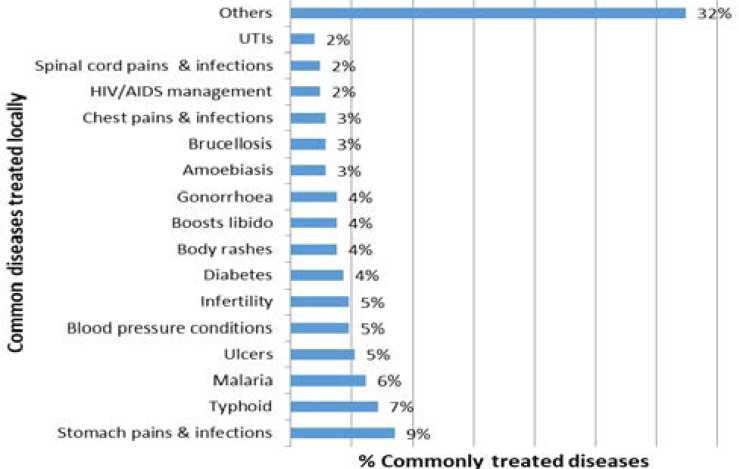
The commonly treated diseases using traded medicinal plants in the Western Kenya TM markets.

Roots (32%) were the commonly traded plant parts followed by barks (24%) and leaves (21%). A combination of twigs and leaves constituted 4% of the most sought medicinal plant parts. Other plant parts that were of importance but less traded included the fruits (1%), tuberous rootstock (1%), flowers (1%), oils (1%) and resins (1%). The commonly traded medicine plant species were invaluable in the treatment of 59 common diseases and socio-cultural ills. The leading disease ailment treated was Stomach-ache and stomach related infections (9%) followed by typhoid (7%), malaria (6%), ulcers (5%), high blood pressure (5%) and infertility (5%). (Figure 2). Following the broader diseases classification using the EBDCS system, it was apparently clear that digestive system and gastrointestinal disorders are the frequently treated disease category followed by genito-urinary infections.

### IUCN Status of frequently traded species

According to the 2001 IUCN Red List categories, version 3.1 , *Warburgia ugandensis* which represented 3% of mostly traded medicinal plant species is classified as Endangered (EN), whereas *Carissa spinarum* (4%) and the Red Stinkwood (*Prunus africana*) were described as Endangered (EN) and Vulnerable (VU) respectively. The leading traded species of *Trichilia emetica* (6%), *Erythrina abyssinica* (3%), *Zanthoxylum chalybeum* (3%), and *Azadirachta indica* (4%) were categorized as of Least Concern (LC).

## Discussion

The basic validation of TM trade can be quantified by the number and presence of experienced and knowledgeable TMPs who have mastered their trade and exude confidence in the eyes of TM clients. The TMPs recorded a mean of 24 years of practice demonstrating how rich the TM markets are with traditional medicine knowledge. A vibrant TM trade and the growing demand for TM presents a picture of the marketability of the medicinal plant species, and also indicate the volume and quantities harvested^20^. The medicine markets represent different cultures and diverse ethnic extractions as demonstrated by the diverse floristic composition of the medicinal plants and their diverse applications^21^.

Men and women TMPs have been found to express specific gender roles. However, women and older participants have demonstrated more know-how in plant use^22^, ^23^ space ordination^23^. Similar research findings have also reported that most TMPs have basic or elementary level of education but display great mastery of TM knowledge^4^. Folk ethno-taxonomy have characterized the TM markets where the locals use vernacular names of the medicinal plant species were commonly used. The main challenge of folk taxonomy is the ever-changing local taxonomy even within a similar ethnic grouping^24^. To mitigate this taxonomic challenge and folk taxonomy variation, authenticated scientific names were applied. The TM industry has grown over the years and has even integrated and naturalized exotic medicinal plant species, for instance, *Azadirachta indica* (native to the Indian subcontinent) and Aloe spp. Recent ethnobotanical study of the Marakwet community of Western Kenya region support the findings that most traditional medicinal plants are used in the treatment of stomach-ache, diarrhoea, chest pains and typhoid^25^. Some research findings have expressed highest informant consensus factor for gastrointestinal system and digestive disorders have also been reported^4^, ^26^.

According to the IUCN Red List database (https://www.iucnredlist.org/species/), Warburgia ugandensis which represented 3% of mostly traded medicinal plant species is classified as Critically Endangered (CR), whereas Carissa spinarum (4%) and the Red Stinkwood (Prunus africana) were described as Endangered (EN) and Vulnerable (VU) respectively. The leading traded species of Trichilia emetica (6%), Erythrina abyssinica (3%), Zanthoxylum chalybeum (3%), and Azadirachta indica (4%) were all found to be of Least Concern (LC). Carissa spinarum shrub has been reported as one of the most frequently traded species in Marsabit and Moyale traditional medicine markets of the Northern Kenya^2^.

## Conclusion

Traditional medicine trade provides a platform of establishing the frequently traded species and the diseases treated. Apparently, the aging TMPs and the reluctance of the younger and educated persons from embracing TM may pose a big challenge in the transfer of TM knowledge. Women practitioners have dominated the trade and the markets have also demonstrated gendered medicine roles. The ranking and prioritization of taxa enables the conservation of the frequently traded medicinal plant species, for instance, Trichilia emetica, Azadirachta indica, Dregea schimperi, Carissa spinarum and the tuberous Tylose ma fassoglense. The increased use of roots and barks and mostly extracted from the tree habit or growth form should be efficiently to guarantee sustainability of the trade and practice. Close attention should be directed to curing digestive and genito-urinary diseases. Finally, the authorities should use sound and credible publications to crack down on the sale of red listed and threatened medicinal plant species.
